# Sepsis secondary to complicated skin and soft tissue infection caused by *Ignatzschineria indica*. First case report in Latin America.

**DOI:** 10.1099/jmmcr.0.005151

**Published:** 2018-04-17

**Authors:** Lucía Cipolla, Laura Derdoy, Daniela Archuby, Adriana Tarzia, Francisco Govedic, Mónica Prieto

**Affiliations:** ^1^​ Servicio Bacteriología Especial, Instituto Nacional de Enfermedades Infecciosas ‘Dr. C. G. Malbrán’, Ciudad Autónoma de Buenos Aires, Argentina; ^2^​ Sección Microbiología, Hospital ‘Dr. J. M. Ramos Mejía’, Buenos Aires, Argentina; ^3^​ Servicio de Infectología, Sanatorio Allende, Córdoba, Argentina

**Keywords:** Ignatzchineria, maggots, 16S rRNA sequencing, sepsis, ciprofloxacin, clindamycin

## Abstract

**Introduction:**

*Ignatzschineria* is a recently recognized genus associated with larvae infestation Members of this genus are pathogens infrequently implicated in human disease. During the last decade, fewer than 10 cases of infection with *Ignatzchineria* species have been reported around the world. Bacteria of the genera *Ignatzchineria* and *Wohlfahrtiimonas* have been isolated from larvae of the parasitic fly *Wohlfahrtia magnifica*, which is found in Europe, Asia and North Africa, and is associated with myiasis in several animal species, but rarely in humans.

**Case presentation:**

We report the first case of sepsis associated with complicated skin and soft tissue infection caused by *I. indica* in Latin America.

**Conclusion:**

The clinical and molecular findings in our report add information to the accumulating data on emerging pathogens of this type, their geographic distribution, the correlation between the emergence of infectious diseases and social and economic inequalities, as well as the effects of global climate changes on potentially unusual distribution of vectors. We consider that fly larvae should be regarded as a potential source of specific arthropod-borne bacterial systemic infections.

## Introduction

The genus *Ignatzschineria* belongs to the class Gammaproteobacteria and comprises at least three species: *Ignatzschineria indica*, *Ignatzschineria larvae* and *Ignatzschineria ureiclastica* [1]. The members of this genus are Gram-negative, aerobic, non-spore forming, non-haemolytic, non-motile, non-pigmented, rod-shaped bacteria [2]. In 2001, Toth *et al*. [3] described the bacterial genus *Schineria,* but 6 years later it was renamed *Ignatzschineria* to honour entomologist Ignatz Rudolph Schiner’s seminal work [4]. Taxonomically this genus is closely related to the genus *Wohlfahrtiim*o*nas*, also a recently recognized genus associated with larvae infestation [5, 6]. Species of the genera *Ignatzchineria* and *Wohlfahrtiimonas* have been isolated from larvae of the parasitic fly *Wohlfahrtia magnifica* [7], which is found in Europe, Asia and North Africa and is associated with myiasis in several animal species, but rarely in humans [1, 8].

Species of the genus *Ignatzschineria* are pathogens infrequently implicated in human disease [9]. In 2007, two cases of bacteraemia due to *I. larvae* were reported, originally identified as *Schineria larvae* [10, 11]. In 2014 two more cases of bacteraemia, clearly associated with maggot infestation due to *I. indica,* were reported [1]. One year later Le Brun *et al*. documented a case of necrotizing wound infection due to *I. ureiclastica* [8]. Subsequently, two more cases have been reported [9, 12].

We report the first case of sepsis in Latin America caused by *I. indica* associated with complicated skin and soft tissue infection, which required lower extremity amputation.

## Case report

In April 2017, a 72-year-old homeless male patient was admitted to the emergency department at the Ramos Mejia Hospital, Ciudad Autónoma de Buenos Aires, Argentina. The patient was in very poor hygienic condition, malnourished and dehydrated. He presented a deep necrotic ulcer in the anterior aspect of his left tibia of 9-months’ evolution, with exposure of both tibia and fibula, complete loss of muscle mass, severe ischaemia, foul discharge and heavy burden myiasis. Unfortunately, maggots had been rapidly discarded, allowing neither bacterial analysis nor entomological identification.

The patient had a history of alcohol abuse and pulmonar tuberculosis in 1980 which resolved after complete treatment.

On physical examination, his blood pressure was 100/60 mmHg, his heart rate 97 and his respiratory rate 20 breaths min^−1^. His body temperature was 36 °C. Haematological and biochemical exams on admission showed: leucocytes 24750 K µl^−1^, with 92.5 % neutrophils; glucose level of 237 mg dl^−1^, haematocrit 40 %, haemoglobin 13.3 g dl^−1^, uraemia 126 mg dl^−1^, creatinine 2017 mg dl^−1^; sodium 127 mmol l^−1^; potassium 5.5 mmol l^−1^; chloride 87 mmol l^−1^.

Two blood culture sets were taken at the time of admission, at two different times. Gram-negative rods were obtained in pure culture. With this preliminary report, the case was described as sepsis caused by skin and soft tissue infection. Intravenous therapy with ciprofloxacin 400 mg/12 h and clindamicin 600 mg/12 h was initiated.

Conventional phenotypic test and MALDI-TOF-MS (Bruker Daltonics) failed to identify the bacteria isolated.

In order to confirm genus and species identification, PCR amplification of the 16S rRNA was performed. The nearly complete sequence of the 16S rRNA gene was amplified by PCR with the conserved primers 8F (5′-AGAGTTTGATYMTGGCTCAG-3′) and 1942R (5′-ACCTTGTTACGACTT-3′), as described previously [13]. The sequence obtained showed a 100 % identity with the sequence corresponding to the 16S RNA ribosomal gene of *I. indica,* type strain FFA1 (GenBank accession number. EU008088.2). The 16S rRNA sequence obtained was deposited in GenBank under number MF062521.

Due to the severity of the lesions, a supracondylar amputation had to be performed in order to allow for adequate and prompt infection source control, and antibiotic treatment for 14 days was completed, which led to resolution of sepsis and normalization of laboratory parameters. The patient had a favourable outcome, with no surgical complications.

Members of the genus *Ignatzschineria* are difficult to identify using traditional methods, including classical biochemical tests and commercial bacterial identification systems. Even MALDI-TOF MS analysis has been unsuccessful. 16S ARNr gene sequencing has proved to be useful for identification, and is currently the most accurate method for clinical diagnostic laboratories.

## Discussion

Our report demonstrates that *I. indica* can cause invasive infection in humans. Since the genus *Schineria*, and actually *I. indica*, have been previously associated only with fly larvae, it is possible that flies attracted to open wounds can cause infestation with larvae infected with bacteria that then spread into the bloodstream.

In Argentina, there are three species of flies that cause myiasis in humans: *Cochliomyia hominivorax*, which is present almost throughout the country, and *Dermatobia hominis* and *Oestrus ovis*, which attack mainly ruminants [14].

*Wohlfahrtia magnifica* is present in Europe, Asia, and North America [8] but it seems that the distribution of this fly is progressively expanding because of its broad adaptation capacities and as a result of climatic changes [15].

In 2011, Almuzara *et al*. described the first case of fulminant sepsis due to *Wolfhartiimonas chitiniclastica* in a homeless patient in Argentina [16]. The patient presented multiple skin lesions in both inguinal regions. Although no larvae were found in the patient's groin lesions, the authors speculated that the skin lesions were infected with the organism which then entered the bloodstream.

Because we could not recover the larvae involved in this case, we could not identify the vector agent. There are no published works related to the study of the microbial flora in native Argentine flies, and therefore we cannot confirm that *I. indica* forms part of the flora of these flies; however, we cannot exclude the possibility. We can speculate about a recent migration of the fly *W. magnifica* to our region or that *I. indica* can colonize the larvae of other parasitic flies associated with myiasis in Argentina. More studies are needed to clarify these issues.

The clinical and molecular findings in our report add information to the accumulating data on emerging pathogens of this type and their geographic distribution. Diseases like myiasis are more prevalent among vulnerable populations, with frequent complications that can even lead to amputation, like the case described in this report.

This case emphasizes the correlation between the emergence of infectious diseases and social and economic inequalities, as well as the influence of global climate changes on potentially unusual distribution of vectors. We consider that fly larvae should be regarded as a potential source of specific arthropod-borne bacterial systemic infections. We highlight the importance of collecting maggots found in patients’ injuries in order to be studied and identified by entomologists, so as to shed light on these emerging pathogens.
